# A phase 3 randomised double-blind placebo-controlled trial of mirtazapine as a pharmacotherapy for methamphetamine use disorder: a study protocol for the Tina Trial

**DOI:** 10.1186/s13063-024-08238-y

**Published:** 2024-06-22

**Authors:** Rebecca McKetin, Tayla J. Degan, Lucy Saunders, Long Nguyen, Gregory Dore, Steven Shoptaw, Michael Farrell, Louisa Degenhardt, Peter J. Kelly, Alyna Turner, Philip J. Clare, Olivia M. Dean, Shalini Arunogiri, Samantha Colledge-Frisby, Juanita Koeijers, David Goodman-Meza, Barbara Sinclair, David Reid, Harry Hill, Jeremy Hayllar, Michael Christmass, Frank Cordaro, Robert Lundin, Willy Liaw, Danica Liu, Ellie Holyoak, Brian Tid-Fung Wu, Joel Keygan, Ava Kontogiannis, Lily Palmer, Caity Morrison, Anna Wrobel, Bec Hyland, Marianne Byrne, Samantha Russell, Emma Zahra, Michael Berk

**Affiliations:** 1 National Drug and Alcohol Research Centre, University of New South Wales, Sydney, Australia; 2 Burnet Institute, Melbourne, Australia; 3 Kirby Institute, University of New South Wales, Sydney, Australia; 4 Department of Family Medicine, David Geffen School of Medicine, Los Angeles, USA; 5 School of Psychology, Faculty of Arts, Social Sciences and Humanities, University of Wollongong, Wollongong, Australia; 6 IMPACT, School of Medicine, Deakin University, Geelong, Australia; 7 Prevention Research Collaboration, The University of Sydney, Sydney, Australia; 8 Alfred Psychiatry Research Centre (MAPrc), Central Clinical School, Monash University, Melbourne, Australia; 9 National Drug Research Institute, Curtin University, Perth, Australia; 10 Center for HIV Identification, Prevention, and Treatment Services, Los Angeles, USA; 11 Illawarra Drug and Alcohol Service, Illawarra Shoalhaven Local Health District, NSW Health, Wollongong, Australia; 12 Drug and Alcohol Services, Barwon Health, Geelong, Australia; 13 Biala City Community Health Centre, Metro North Health, Brisbane, Australia; 14 Next Step Community Alcohol and Drug Service East Perth, East Perth, Australia; 15 Drug and Alcohol Services of South Australia, Adelaide, Australia; 16 Alcohol, Tobacco & Other Drug Services, Townsville, Australia; 17 Grampians Health, Ballarat, Australia; 18 Florey Institute for Neuroscience and Mental Health, University of Melbourne, Melbourne, Australia

**Keywords:** Substance use disorders, Methamphetamine, Mirtazapine, Clinical trial, Depression, Sleep, Pharmacotherapy, Treatment, Psychiatry, Addiction

## Abstract

**Background:**

There are no approved pharmacotherapies for methamphetamine use disorder. Two preliminary phase 2 randomised controlled trials have found mirtazapine, a tetracyclic antidepressant, to be effective in reducing methamphetamine use. The proposed Tina Trial is the first phase 3 placebo-controlled randomised trial to examine the effectiveness and safety of mirtazapine as an outpatient pharmacotherapy for methamphetamine use disorder.

**Methods:**

This is a multi-site phase 3 randomised, double-blind, placebo-controlled parallel trial. Participants are randomly allocated (1:1) to receive either mirtazapine (30 mg/day for 12 weeks) or matched placebo, delivered as a take-home medication. The target population is 340 people aged 18–65 years who have moderate to severe methamphetamine use disorder. The trial is being conducted through outpatient alcohol and other drug treatment clinics in Australia. The primary outcome is measured as self-reported days of methamphetamine use in the past 4 weeks at week 12. Secondary outcomes are methamphetamine-negative oral fluid samples, depressive symptoms, sleep quality, HIV risk behaviour and quality of life. Other outcomes include safety (adverse events), tolerability, and health service use. Medication adherence is being monitored using MEMS® Smart Caps fitted to medication bottles.

**Discussion:**

This trial will provide information on the safety and effectiveness of mirtazapine as a pharmacotherapy for methamphetamine use disorder when delivered as an outpatient medication in routine clinical practice. If found to be safe and effective, this trial will support an application for methamphetamine use disorder to be included as a therapeutic indication for the prescription of mirtazapine.

**Trial registration:**

Australian and New Zealand Clinical Trials Registry ACTRN12622000235707. Registered on February 9, 2022.

## Introduction

### Background and rationale {6a}

Methamphetamine (also known as “crystal meth” or “ice”) is a significant and growing global public health concern with an estimated 7.4 million people worldwide dependent on the drug [1, 2]. Methamphetamine use disorder is a chronic, relapsing condition [3] that is associated with elevated mortality, increased incidence of HIV and hepatitis C infection, poor mental health (suicidality, psychosis, depression, and violence), and increased risk of cardiovascular events [4]. Use accounts for a significant excess number of psychiatric hospital admissions and emergency department presentations in Australia [5]. In the USA, methamphetamine is increasingly used in conjunction with opioids, and this is contributing to the growing number of opioid-related overdose deaths [6].

Currently, there are no approved pharmacotherapies to assist with the treatment of methamphetamine use disorder [7, 8]. Although individual trials have found positive results (e.g., long-acting slow-release amphetamine preparations [9] and a combination of naloxone and bupropion [10]), many promising options have failed to produce the expected reductions in methamphetamine use [11, 12], and there remains insufficient evidence to support any pharmacotherapy option for methamphetamine use disorder [13]. Effective pharmacotherapy has the potential to dramatically increase treatment coverage, enhance treatment engagement and retention, and mitigate against poor health outcomes for people with methamphetamine use disorder.

One promising pharmacotherapy candidate is mirtazapine, with significant reductions in methamphetamine use found in two preliminary phase 2 trials in the USA [14, 15]. The first trial (*N* = 60) found that 12 weeks of mirtazapine (30 mg/day) significantly reduced methamphetamine use relative to placebo amongst sexually active men who have sex with men [14]. The second trial replicated these positive results after 24 weeks of mirtazapine treatment with a larger sample of cisgender men, transgender men, and transgender women, who have sex with men (*N* = 120) (positive urine test results at 24 weeks relative to placebo 63% vs. 74%; RR 0.75, 95% CI 0.56–1.00, *p* = 0.04) [15]. In the second trial, mirtazapine also reduced depressive symptoms and improved sleep [15]. A meta-analysis of outcomes for these trials showed consistent signals for mirtazapine for the reduction of methamphetamine use, though not for reducing depressive symptoms [16]. The consistent signal across these trials for reducing methamphetamine indicates a need for a phase 3 trial to demonstrate the benefits of mirtazapine when used in a broader population in routine clinical practice.

### Objectives {7}

The aim of this trial (the Tina Trial) is to establish the effectiveness and safety of mirtazapine as an outpatient pharmacotherapy for methamphetamine use disorder in routine clinical practice in an Australian setting.

The primary hypothesis is that oral mirtazapine (30 mg/day) from baseline to 12 weeks will, compared to placebo, reduce self-reported days of methamphetamine use.

The secondary hypotheses are that oral mirtazapine (30 mg/day) for 12 weeks will, compared to placebo:

- Increase abstinence from methamphetamine use, as assessed by methamphetamine-negative oral fluid samples,
- Reduce depressive symptoms,
- Improve sleep quality,
- Reduce HIV risk behaviour, and
- Improve quality of life.

### Trial design {8}

The Tina Trial is a multi-site double-blind randomised placebo-controlled parallel group, two-arm, superiority trial with a 1:1 allocation ratio. The protocol adheres to the Standard Protocol Items: Recommendations for Interventional Trials (SPIRIT) Statement [17]. The protocol was prospectively registered with the Australian New Zealand Clinical Trials Registry on February 9th 2022 (ACTRN12622000235707). The universal trial number is U1111-1271–8220.

## Methods: participants, interventions and outcomes

### Study setting {9}

The study is being conducted via alcohol and other drug outpatient services in Australia. Sites include the Illawarra Drug and Alcohol Services in Wollongong (New South Wales); the Mental Health, Drugs and Alcohol Services, Barwon Health in Geelong (Victoria); Biala City Community Health Centre, Brisbane (Queensland); the Next Step Community Alcohol and Drug Service in Perth (Western Australia) and the Alcohol, Tobacco & Other Drug Services in Townsville (Queensland).

### Eligibility criteria {10}

Participants must be aged 18–65 years, meet Diagnostic and Statistical Manual of Mental Disorders (5th edition; DSM-5) [18] criteria for moderate to severe methamphetamine use disorder in the past year, and be currently using methamphetamine (using methamphetamine at least twice weekly, based on self-report, and have a positive drug screen for the use of amphetamines). Participants must also be willing to use effective contraception (women only), provide contact details for follow-up, provide contact details for a treating physician (which can be the trial site physician), and be able to provide informed consent and comply with the study protocol.

Participants will be ineligible if they:

- Need acute care (e.g., acute suicidality or psychosis, require medical detoxification)
- Have attempted suicide within the past year,
- Are incarcerated,
- Are in inpatient treatment (including residential rehabilitation and inpatient detoxification^1^),
- Are taking prescribed anti-depressant medication,
- Have taken monoamine oxidase inhibitors in the past 14 days,^2^
- Have contraindications for mirtazapine or are at a high risk for adverse reactions to mirtazapine,
- Have galactose intolerance, Lapp lactase deficiency or glucose-galactose malabsorption (lactose is an excipient in the trial medication),
- Are pregnant or lactating,
- Are unwilling or unable to avoid pregnancy during the trial, or
- Are participating in another clinical trial.

### Who will take informed consent? {26a}

Participant consent is obtained by the trial research team at the start of the eligibility assessment. During the eligibility medical screen, the trial physician confirms that the participant is eligible, that they can provide informed consent, and they answer any questions that the participant has about the trial procedures. Consent can be either written, verbal or obtained electronically.

### Additional consent provisions for collection and use of participant data and biological specimens {26b}

This trial does not involve storing biological specimens for use in ancillary studies. All biological specimens collected for the trial will be destroyed 3 months after use.

Participant data may be shared in a de-identified form with other researchers for research purposes. This requires written permission from the Sponsor. All relevant ethics approvals must be upheld, and the source of the data and the funding body must be acknowledged. De-identified data may be stored on a public repository for the purposes of data sharing in a way that participants cannot be identified. Identifiable participant data can only be shared with the written permission of the Sponsor and appropriate local ethics and governance approvals.

## Interventions

### Explanation for the choice of comparators {6b}

The comparator (placebo) has been chosen because there are currently no approved medications for methamphetamine use disorder.

### Intervention description {11a}

The intervention is daily oral mirtazapine 30 mg per day, or matched placebo, for 12 weeks, taken as one tablet each evening. This is provided in bottles of 35 tablets at baseline, week 4 and week 8. A tapering dose of 15 mg per day, for 28 days, is provided at week 12. All participants are provided with a self-help booklet (“*On Ice*”, National Drug and Alcohol Research Centre, University of New South Wales), referral information for local health services, and have access to usual care during the study.

### Criteria for discontinuing or modifying allocated interventions {11b}

The participants are free to discontinue the trial medication at any time. The trial medication may be temporarily discontinued by the study team (e.g., to monitor an adverse event or suspected pregnancy). The study team may permanently discontinue a participant from the trial medication if they become pregnant, develop contraindications for mirtazapine, are diagnosed with a medical condition that significantly increases their risk of AEs, or are unable to comply with the study protocol to an extent that significantly compromises their safety (e.g., unable to attend medical assessments). Participants may be temporarily or permanently changed to a 15-mg dose of the trial medication in response to adverse reactions to the trial medication. Participants who are discontinued from the medication remain in the study to complete study assessments (unless they are also withdrawn from the study or withdraw their consent to participate).

Participants are free to withdraw from the study at any time. The study team may withdraw participants from the study if:

- The participant is unable to complete study procedures (e.g., becomes incarcerated),
- There is significant non-compliance with the study protocol and/or behaviour that compromises the safety and wellbeing of the trial participant or the trial personnel, or
- If the participant meets an exclusion criterion that precludes further study participation.

If a participant withdraws from the study, or is withdrawn by the study team, they are not followed up for further assessments. However, they will be asked to attend a final medical assessment to review AEs and to return their study medication.

### Strategies to improve adherence to interventions {11c}

Adherence to the trial medication (baseline to week 12) is monitored using a medication event monitoring system, MEMS® Smart Caps, which record the time and date of each bottle opening. Adherence data is reviewed at week 4 and week 8, and participants are counselled on adherence strategies. An adherent dose is defined as any bottle opening in a 24-h period (ending at 3 am) with no penalty for multiple openings. MEMS® Smart Caps® also display the number of bottle openings each day so that participants can see when they have taken their daily dose.

### Relevant concomitant care permitted or prohibited during the trial {11d}

Participants may engage in treatment as usual throughout the trial. Where this precludes participation in the trial (either for logistical or safety reasons) participants may be discontinued from the trial medication or withdrawn from the study.

### Provisions for post-trial care {30}

At the end of the trial, all participants are offered a referral to substance use treatment, including a referral for the ongoing prescription of mirtazapine. Participants who suffer harm from trial participation can seek compensation in accordance with the Medicines Australia Compensation Guidelines (https://medicinesaustralia.com.au/policy/clinical-trials/indemity-and-compensation-guidelines/).

### Outcomes {12}

#### Primary outcome

Reported days of methamphetamine use: The primary outcome is a change in self-reported days of methamphetamine use in the past 4 weeks from baseline to week 12. Self-reported days of methamphetamine use are assessed using the Timeline Followback (TLFB) [19]. The TLFB is a retrospective self-report measure that uses a calendar to aid recall. It has 88% sensitivity and 96% specificity against amphetamine urine test results, and 0.77 test–retest agreement [20]. TLFB data will be validated against biologically verified abstinence from methamphetamine use.

#### Secondary outcomes

*Abstinence from methamphetamine use*: Methamphetamine-negative oral fluid samples (< 25 ng/mL methamphetamine) taken at weeks 4, 8 and 12 using a commercial oral fluid collection device. Oral fluid is a sensitive and stable medium for the detection of methamphetamine and correlates highly with plasma tests [21].

*Depressive symptoms*: Total score on the Patient Health Questionnaire-9 (PHQ-9) [22] at week 12. The PHQ-9 is a brief 9-item questionnaire that has excellent internal consistency (Cronbach’s alpha of 0.89), test–retest reliability (*r* = 0.84) and good construct validity against other measures [22].

*Sleep quality*: Total score on the Athens Insomnia Scale (AIS) [23] at week 12. The AIS is an 8-item self-report measure which has 0.9 test–retest reliability and has been validated against other measures of sleep quality [23].

*HIV risk behaviour*: Total score on a modified version of the HIV Risk-taking Behaviour Scale (HRBS) from the Opioid Treatment Index [24] at week 12. The HRBS is a validated and reliable scale that provides a composite risk index for injecting and sexual behaviour. The modified version of this scale is available from the authors on request.

*Quality of life*: The utility score on the EuroQol-5D-5L (EQ-5D) [25] at week 12 is used to measure quality of life.

#### Tertiary/exploratory outcomes

*Suicidality*: Scores of 3 or greater on the Columbia Suicide Severity Rating Scale Screener (CSSRS-S) [26, 27] at any time in the 12-week active medication phase. A score of 3 or more on the CSSRS-S predicts a significant increase in the risk of a subsequent suicide attempt [27].

*Other substance use*: Total days of use for other drug classes (tobacco, alcohol, cannabis, cocaine, ecstasy, hallucinogens, inhalants, and heroin) in the 4 weeks prior to the week 12 assessment.

*Treatment satisfaction*: Scores on the Treatment Satisfaction Questionnaire for Medication Version II (TSQM II) [28] at week 12 are being used to assess treatment satisfaction. The TSQM II provides a global satisfaction summary score and subscale scores for medication convenience, side effects, and effectiveness. Additional questions have been included at other weeks to assess tolerability and expected reactions to mirtazapine.

*Anxiety*: Total scores on the Generalized Anxiety Disorder – 7 Item (GAD-7) [29] at week 12. The GAD-7 is a validated screening tool for generalised anxiety disorder that can also be used to assess the severity of anxiety [29].

*Participants’ impression of their health status*: The score on the single-item Patient Global Impression – Improvement (PGI—I) [30] is used at week 12 to assess whether participants perceive that their health status has improved since the start of the trial.

*Concomitant medications*: All medications taken by participants during the trial are recorded on a template adapted from the National Institute of Health Concomitant Medications Form [31].

*Health economics*: Health economics data collected include the EuroQol 5D [25] and the Work Productivity and Activity Impairment Questionnaire – General Health (WPAIQ-GH) V2 [32] at week 12, and contact with health services and the criminal justice system from baseline to the week 12 assessment.

*Adverse events (AEs):* The percentage of participants reporting AEs, and serious adverse events (SAEs), by System Organ Classification, will be coded according to the Medical Dictionary for Regulatory Activities (MedDRA) [33]. AEs will be counted once only for a given participant.

### Participant timeline {13}

**Fig. 1:**
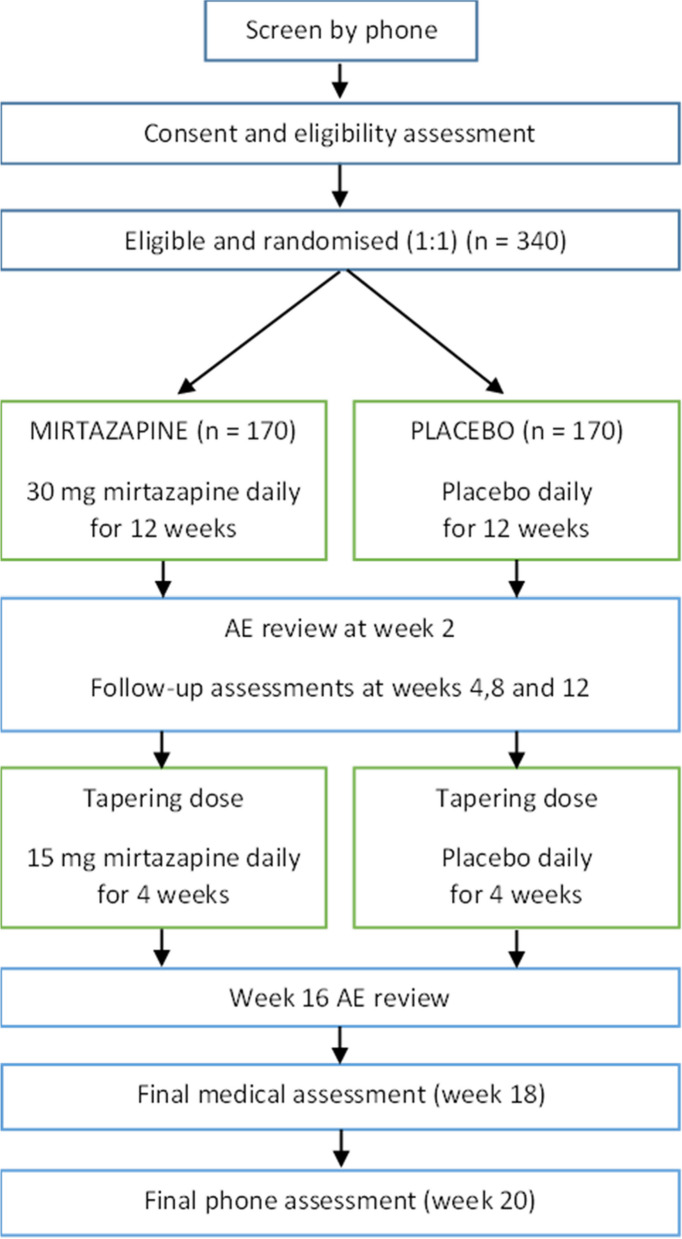
Flow diagram for the trial

All participants undergo an initial phone screening and eligibility assessment, after which they have evaluation assessments at baseline, week 4, week 8, week 12, and week 20. They also have an initial medical screening (as part of the eligibility assessment), a final medical review (week 18), and two additional phone follow-ups to review AEs at weeks 2 and 16. The detailed schedule for assessments is provided in Table 1, and the study flow chart is presented in Fig. 1.

**Table 1:**
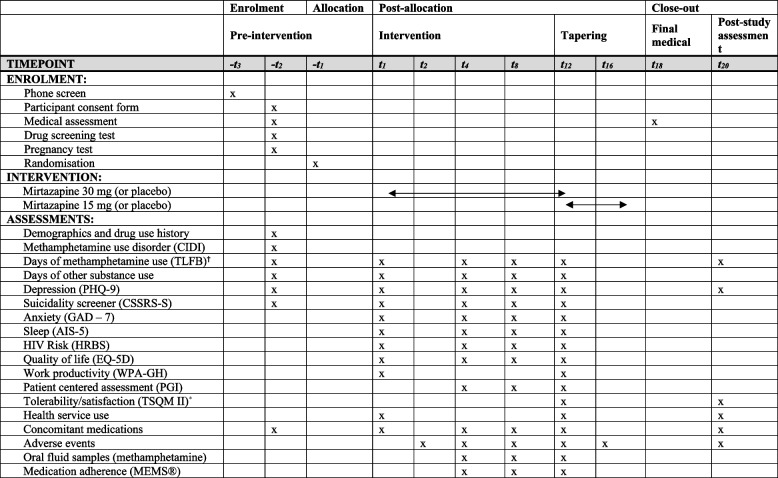
Schedule of enrolment, interventions and assessments

*TLFB* Timeline Followback, *PHQ-9* Patient Health Questionnaire–9, *AIS* Athens Insomnia Scale, *HRBS* HIV Risk Behaviour Scale, *EQ-5D* Euroqol 5D, *CSSRSS* Columbia Suicide Severity Risk Scale Screener, *PGI* Patient Global Impression, *CIDI* Composite International Diagnostic Interview modified to capture a DSM 5 methamphetamine use disorder, *GAD-7* the Generalised Anxiety Disorder 7 item scale, *WPAI-GH* Work Productivity and Activity Impairment Questionnaire – General Health V2, *TSQM II* Treatment Satisfaction Questionnaire for Medication Version II

^*^The TSQM II will be substituted with the unpublished questions on medication tolerability at weeks 4 and 8

^**†**^28 days are provided for screening and eligibility assessments prior to randomisation; timepoints are indicative only All assessments should be scheduled no sooner than 7 days after the previous assessment

### Sample size {14}

The sample size (*N* = 340; 170 per group) will enable us to detect a minimum rate ratio of 0.75 on our primary outcome (equivalent to a reduction from 25 days of methamphetamine use in the past 4 weeks at baseline to 20 days of use in the past 4 weeks at week 12) with 90% power (two-tailed test, *p* = 0.05). This sample size calculation is based on the effect size found in the most recent trial of mirtazapine for methamphetamine dependence [15] and allows for up to 25% attrition.

### Recruitment {15}

Participants are being recruited from the community via advertisements (e.g., local newspapers, free press, and social media), flyers (e.g., placed in needle and syringe programmes, community health care centres) and word of mouth. Participants can also be referred from helplines, health services, and other research studies.

## Assignment of interventions: allocation

### Sequence generation {16a}

Eligible participants will be randomly assigned (1:1) to receive either placebo or mirtazapine based on a computer-generated permutated block randomisation sequence with variable block sizes, stratified by site, sex (male vs. female or other) and depression (PHQ-9 score < 10 vs. 10 or greater) assessed at eligibility.

### Concealment mechanism {16b}

Medication packaging and pill appearance in each arm are identical to conceal treatment allocation. A removable adhesive label indicates whether the medication is placebo or mirtazapine. This is removed by the pharmacy prior to dispensing.

### Implementation {16c}

The Data and Safety Monitoring Board (DSMB) statistician generates the randomisation schedule and sends this to the trial site pharmacy. The generated randomisation schedule includes a list of unique study identifiers and their corresponding condition allocation. The pharmacy staff allocate each participant’s condition based on their unique study identifier, which is provided by the study team. The unique study identifier is a 6-digit number indicating the randomisation strata (trial site, sex, depression) and the sequence number within the randomisation strata.

## Assignment of tnterventions: blinding

### Who will be blinded {17a}

All trial participants, trial staff, trial investigators, care providers, and the trial statistician are blind to the assignment of the intervention. The only people who know the condition allocation are the DSMB statistician and the trial site pharmacy staff. A statistical analysis plan will be finalised prior to unblinding, and the analysis will be blind to condition.

### Procedure for unblinding if needed {17b}

Unblinding can occur when knowing condition allocation is necessary to make clinical treatment decisions (e.g., in a medical emergency, pregnancy) or at the request of the DSMB. Unblinding is at the discretion of either the DSMB, the trial investigators, or the treating physician in an emergency. Unblinding can be done either via an online portal or via the trial site pharmacy.

## Data collection and management

### Plans for assessment and collection of outcomes {18a}

Data are collected on an electronic Case Report Form (eCRF) implemented using Research Electronic Data Capture (REDCap) [34, 35]. Each eCRF is manually reviewed by data monitoring personnel for accuracy and completeness. The recorded TLFB data on days of methamphetamine use (the primary outcome measure) are reconciled against auto-calculated fields in REDCap. These auto-calculated fields count the days between the start and end dates in the TLFB calendar, and the number of days of methamphetamine use recorded within these dates. Data monitoring personnel review and resolve any discrepancies between the auto-calculated field and the manually entered data. All medication coding and MedDRA coding are done by trained data monitoring personnel to maintain consistency. All trial researchers receive training in the administration of data collection instruments and navigating the eCRF in REDCap. Weekly meetings between the trial researchers and the trial management team are used to review procedures and identify and rectify any data collection issues.

### Plans to promote participant retention and complete follow-up {18b}

Participant retention and follow-up is enhanced by obtaining and maintaining comprehensive contact information for participants, reimbursing participants AUD50 per assessment, and providing SMS text and/or phone call reminders to participants 1–3 days prior to their next scheduled assessment. Participants are also reimbursed AUD10 per medication bottle return to enhance return of MEMS® Smart Caps. We additionally have implemented field interviewing procedures used in sentinel surveys of people who use drugs [36]. This maximises participant follow-up by allowing assessments to be carried out at a location convenient to the participant.

### Data management {19}

Data are being collected via a secure password-protected data platform (REDCap [34, 35]) maintained by the University of New South Wales. Access is password-protected and requires two-factor authentication. Data access groups are assigned to users in REDCap to ensure appropriate data access for study personnel. Paper and other electronic records containing identifiable data are stored securely at the trial site (i.e., locked filing cabinet/password-protected file) and stored separately to trial outcomes data. At the close of the study, data will be managed according to the University of New South Wales data storage and retention policies. The data will be maintained for at least 15 years, as recommended by the National Health and Medical Research Council of Australia, after which it will be destroyed.

### Confidentiality {27}

All participants are assigned a unique study identifier on screening which is used to de-identify data. Only authorised study personnel have access to personally identifiable data. Identifiable data are only disclosed to third parties with the permission of the participant or as required by law. Study data may be shared with third parties for the purpose of research, but only in a de-identified format. Trial data will be reported in a way that does not identify any individual participant in the study. For further details, see the “Data Management {19}” section.

### Plans for collection, laboratory evaluation and storage of biological specimens for genetic or molecular analysis in this trial/future use {33}

There are no plans for future genetic or molecular analysis of biological samples collected in this study. De-identified oral fluid samples are sent to the Victorian Institute of Forensic Medicine for analysis and are subsequently destroyed. Other biological tests (pregnancy tests and drug screening tests) are disposed of immediately after the results have been recorded in the eCRF.

## Statistical methods

### Statistical methods for primary and secondary outcomes {20a}

Descriptive statistics will be presented as the mean (standard deviation) for continuous parametric measures and median (inter-quartile range) for highly skewed measures. Categorical variables will be presented as a percentage per category. Baseline descriptive statistics will be compared between the treatment conditions using appropriate inferential statistics.

The main analysis of both the primary and secondary endpoints will be based on the intention-to-treat population (defined as participants who took at least one dose of medication) and based on unimputed data. All tests will be two-sided with *p* < 0.05.

#### Analysis of the primary outcome

The main effect of medication on days of methamphetamine use will be tested using a mixed model with a group (placebo vs. mirtazapine) by time (baseline, week 4, week 8, week 12) interaction effect, with time entered as a factor variable, producing individual effect estimates for each time point and making no assumptions about the linearity of changes over time. The primary timepoint is week 12. Random intercept terms will be included in the model to account for clustering on repeats and by site (37).

#### Analysis of the secondary outcomes

The analyses of the methamphetamine-negative oral fluid samples (no [0], yes [1] at weeks 4, 8 and 12) will be tested using a mixed model with a group contrast (placebo vs. mirtazapine) across all follow-up time points to obtain an average treatment effect. The effect of mirtazapine on other secondary endpoints (depression, sleep, quality of life and HIV risk) will be tested using the same approach as used for the primary outcome. That is, a mixed model using a group (placebo vs. mirtazapine) by time (baseline vs. follow-up [weeks 4, 8 and 12]) with time entered as a factor variable, producing individual effect estimates for each time point and making no assumptions about the linearity of changes over time. The primary timepoint for these endpoints is week 12.

### Interim analyses {21b}

Interim analysis of the data is not planned.

### Methods for additional analyses (e.g., subgroup analyses) {20b}

Subgroup analyses may be undertaken for (a) men vs. women, and (b) co-occurring depression (based on a PHQ-9 score of < 10 vs. 10 or greater). Post hoc power analysis will guide the interpretation of these analyses and whether they are viable.

### Methods in analysis to handle protocol non-adherence and any statistical methods to handle missing data {20c}

Sensitivity analyses will be undertaken that impute missing data. A per-protocol analysis will be undertaken that excludes participants who did not adhere to the protocol. The definition of the per-protocol population, and any methods for data imputations, will be provided a priori in the statistical analysis plan. The statistical analysis plan will be published on the Australian and New Zealand Clinical Trials Registry (ACTRN12622000235707) prior to unblinding.

### Plans to give access to full protocol, participant-level data and statistical code {31c}

De-identified data, study protocol and data dictionary will be available from 12 months after the end date of the study. De-identified data may be provided by study investigators to third parties for the purposes of research, along with the full study protocol, on application to the Sponsor (UNSW), provided that this is in accordance with ethics approvals. Statistical code for published analysis will be made available along with any related publications at the request of the publisher.

## Oversight and monitoring

### Composition of the coordinating centre and trial steering committee {5d}

The coordinating centre is the National Drug and Alcohol Research Centre at the University of New South Wales, Sydney, Australia. The trial steering committee (known as the Trial Management Group) is comprised of the study investigators, who meet monthly throughout the trial to monitor progress. Staff from the coordinating centre meet weekly with the research team to monitor data collection.

### Composition of the data monitoring committee, its role and reporting structure {21a}

Oversight is by an independent DSMB comprised of members with content expertise, clinical trial experience, lived experience and a statistician. The DSMB meet by teleconference at least bi-annually throughout the trial and report to the study Sponsor. The DMSB operate under a charter which they review and approve (the DSMB Charter can be obtained from the study Sponsor on request).

### Adverse event reporting and harms {22}

Information on AEs is solicited from participants by the trial researchers at each assessment using open-ended questions. Information recorded on the eCRF about each AE includes onset, completion, severity, relatedness to the study medication, and whether the AE is serious (i.e., an SAE). AEs recorded in REDCap are reviewed weekly by the study physician (a licensed medical practitioner), who confirms severity and relatedness, and who can request a medical assessment with the study participant to review the AE. All AEs are reviewed by the DSMB biannually. All AEs are followed for outcome information until resolution or stabilisation. SAEs are reported to the study Sponsor within 24 h, and to the DSMB within 72 h. All unexpected serious adverse reactions are reported to the Therapeutic Goods Administration within 7 days (fatal or life-threatening) or 15 days (non-life threatening) of the study team becoming aware of the event. SAEs are reported to the governing ethics committees as required.

All AEs are coded by System Organ Class (SOC) based on MedDRA. Safety analyses will report on the number and percentage of participants reporting AEs and SAEs in each treatment condition, by SOC. An AE will be counted only once for a given participant, with the event counted being the most severe.

### Frequency and plans for auditing trial conduct {23}

The coordinating centre, on behalf of the Sponsor (the University of New South Wales), conducts site initiation visits prior to the start of participant recruitment, six-monthly site monitoring visit during the trial conduct, and site-close out visits at the end of the study. Annual progress reports, and a final study report, are submitted to the governing Human Research Ethics Committee.

### Plans for communicating important protocol amendments to relevant parties (e.g., trial participants, ethical committees) {25}

Amendments to the research protocol are submitted to the governing ethics committee for approval, and subsequently to all site-specific governance authorities, prior to implementation. Approved changes are implemented via updating the eCRF and the trial master file, and direct communication with study investigators and relevant study staff. Substantive changes to the trial protocol will be posted on the Australian New Zealand Clinical Trials Registry (ACTRN12622000235707). Participants are notified of any changes to the protocol, or new information, that impacts on their safety or wellbeing or the ethical acceptability of the trial.

### Dissemination plans {31a}

Findings will be disseminated via journal publications, conferences, seminars, the trial website (tinatrial.info), and they will be posted on the ANZCTR website. With their permission, participants will be sent a report summarising the study outcomes and a summary of their own study data.

## Discussion

If mirtazapine is found to be safe and effective, this phase 3 trial will provide the needed evidence to support the use of mirtazapine as a pharmacotherapy for methamphetamine use disorder. Given the current lack of approved medications for treating methamphetamine use disorder [4], identifying an effective pharmacotherapy could be transformative. Our pragmatic and inclusive trial design will establish realistic treatment effects. We also have the potential to identify a possible mechanism of action (e.g., whether any benefits of mirtazapine are mediated by reductions in depression or improvements in sleep). Information pointing to a mechanism of action provides further justification for the wide-scale uptake of mirtazapine if it is found to be effective. As an already available and affordable take-home generic medication in most countries, the uptake of mirtazapine in clinical practice could be swift and could dramatically increase treatment coverage for methamphetamine use disorder.

Conversely, any lack of benefit found, or concerns with safety that are uncovered, will also be clinically important. Mirtazapine is already being used off-label to treat methamphetamine use disorder in many settings, due to the developing evidence base coupled with the lack of alternative approved medications. Such prescribing practices, particularly when they occur outside of the regulation of a clinical trial, may be liable to additional safety concerns (e.g., increased risk of overdose or suicidality). Moreover, the benefits of mirtazapine observed in previous phase 2 trials were modest (15–18% reductions in methamphetamine use (14, 15)). This trial will confirm the existence and magnitude of any benefits, and provide safety information, in a larger, broader sample, where prescribing is occurring in a manner akin to routine clinical practice.

We also anticipate that the Tina Trial will foster interest and capacity in pharmacotherapy trials for methamphetamine use disorder in Australia. Not only is the trial team working with various frontline clinical services to carry out trial recruitment, but we have also adapted trial methods to be more amenable to the target population. This has included ensuring that assessment methods and advertising strategies are designed with the input of people who have lived experience of using methamphetamine, ensuring that assessments are not overly burdensome, and that trial researchers receive training and support on interviewing people who use illicit drugs. We have also gone to great lengths to develop recruitment methods that reach beyond people who are already engaged with clinical services (e.g., advertising on social media) and we have implemented field interviewing protocols to allow interviews to take place at a location convenient to the participant when they are unable or disinclined to attend trial site clinics in person. We remunerate participants in line with our national research guidelines on consumer engagement, and we actively engage with people who have lived experience, including having representation on our investigator team and on our DSMB. Critical to the success of the trial is having a dedicated research officer at each trial site to concierge both clinic staff and participants through the clinical trial process. This has helped overcome resource constraints at health services and bolstered the confidence of clinicians in carrying out trial-related duties. Regardless of the findings of this trial, we hope that the Tina Trial will embed experience and procedures that will provide a platform for future clinical trials in this area.

## Trial status

The current Human Research Ethics Committee-approved protocol is version 9.0 (11 March 2024). The first participant was randomised on 17th November 2022. On May 15, 2024, 239 participants had been randomised. It is anticipated that recruitment (*N* = 340) will be completed by mid-2025.

## Data Availability

The datasets generated during the trial are not publicly available due to privacy laws but de-identified data can be made available on reasonable request to the Sponsor, pending appropriate ethics approval and the approval of the investigator team.

## Abbreviations

AE: Adverse event
AIS: Athens Insomnia Scale
CSSRS-S: Columbia Suicide Severity Rating Scale Screener
DSMB: Data and Safety Monitoring Board
DSM: Diagnostic and Statistical Manual of Mental Disorders
eCRF: Electronic case report form
EQ-5D: EuroQol—Version 5 – 5 level
GAD: Generalised anxiety disorder
HRBS: HIV Risk-taking Behaviour Scale
MeDRA: Medical Dictionary for Regulatory Activities
MEMS: Medication event monitoring system
NHMRC: National Health and Medical Research Council
PGI—I: Patient Global Impression – Improvement
PHQ: Patient Health Questionnaire
REDCap: Research Electronic Data Capture
RR: Rate ratio
SAE: Serious adverse event
TLFB: Timeline Followback
TSQM II: Treatment Satisfaction Questionnaire for Medication Version II (
WPAI-GH: Work Productivity and Activity Impairment Questionnaire – General Health

## Footnotes

1 This relates to the status of the participant at trial enrolment and does not preclude the participant from receiving these treatments during the trial.

2 This requirement applies to the 14 days prior to starting the trial medication.
